# De novo cancer-related mortality after solid organ transplantation in England: the EpCOT study

**DOI:** 10.1038/s41416-026-03529-4

**Published:** 2026-07-01

**Authors:** Charlotte Stephens, David Winter, Mike Hawkins, Raoul Reulen, Adnan Sharif

**Affiliations:** 1 Department of Nephrology and Transplantation, University Hospital Birmingham, Birmingham, UK; 2 Institute of Immunology and Immunotherapy, University of Birmingham, Birmingham, UK; 3 Applied Health Sciences, University of Birmingham, Birmingham, UK

**Keywords:** Cancer epidemiology, Cancer screening

## Abstract

**Background:**

Cancer is a major cause of mortality after solid organ transplantation (SOT). Quantifying this risk is difficult due to heterogeneous reports and methodological limitations.

**Methods:**

We conducted a population-based cohort study linking data between the UK Transplant Registry, National Cancer Registry, Civil Registration of Deaths, and Hospital Episode Statistics for all SOT recipients in England between 1st June 1992 and 31st December 2015.

**Findings:**

Among 50,762 SOT recipients, 22,361 deaths were recorded, of which 3550 (15.9%) were due to cancer, making it the second leading cause of mortality. Overall cancer (excluding non-melanomatous skin cancer) standardised mortality ratio (SMR) was 2.12 (95% CI: 2.05–2.19). Cancer mortality was highest among lung recipients (SMR 3.49; 95% CI: 2.96–4.06), and lowest in kidney recipients (SMR 2.00; 95% CI: 1.92–2.08). Non-Hodgkin’s Lymphoma had highest mortality after SOT (SMR 9.86; 95% CI: 9.01–10.75) and was elevated across all transplant types. However, liver and kidney cancer mortality was disproportionately elevated among liver (SMR: 9.30; 95% CI: 7.53–11.24) and kidney transplant recipients (SMR: 5.11; 95% CI: 4.34–5.93), respectively. Several cancers showed no increased mortality.

**Interpretation:**

De novo cancer remains a major cause of long-term mortality after SOT in England, with risk stratified by cancer and/or allograft.

## Introduction

Solid organ transplantation (SOT) is a lifesaving or life-enhancing intervention for patients with end-stage organ failure [1]. Globally, 172,409 transplant procedures were documented as performed in 2023 [2], and over a million people currently live with an allograft. Balanced against transplantation survival benefits, a lifelong requirement for anti-rejection therapy introduces susceptibility to immunosuppression-related complications such as cancer [3]. The underlying pathophysiology may relate to increased cumulative exposure to the direct oncogenic effects of certain immunosuppressive agents, an inflammatory milieu associated with organ failure, and increased susceptibility to oncogenic infections, frequently on a background of comorbidities associated with elevated cancer risk.

While cancer incidence is elevated for SOT recipients, that may not translate directly to elevated cancer-related mortality. SOT recipients have increased all-cause mortality versus the general population [4], with several competing risks for mortality including infection, cardiovascular disease or allograft failure. Previous work published over a decade ago demonstrated cancer-related mortality risk difference in England was higher after kidney transplantation versus the general population [5], However, this data relied upon hospital administration data alone which has several methodological limitations [6]. It is imperative we improve our understanding of post-transplant cancer mortality risk to ensure appropriate counselling before transplantation and encourage evidence-based management post-transplantation. Globally, several studies have reported elevated cancer mortality risk versus the general population. However, the magnitude of risk is heterogenous and ranges from 1.4 in South Korea [7] up to 2.9 in Australia and New Zealand [8]. This variation highlights differences in study methodology, cancer surveillance practices, population demographics, healthcare access and/or immunosuppression protocols between countries. Therefore, obtaining data specifically for a United Kingdom cohort is important.

In this population-cohort study, we aim to explore cancer-related mortality among SOT recipients in England to better characterise mortality risk after development of de novo post-transplantation cancer.

## Methods

### Data linkage

This retrospective, population-based, record-linked registry study includes all SOT performed in England between 1st January 1995 until 31st December 2015. Recipients were followed up for cancer outcomes until 31st December 2017 and for mortality outcomes until 31st December 2022. Data linkage was conducted by NHS Digital (now NHS England) using identifiable patient information, including NHS number, date of birth, sex, and postcode. Linkage was facilitated between the following national databases:

1. *United Kingdom Transplant Registry (UKTR)*. Maintained by NHS Blood and Transplant (NHSBT), this registry receives mandatory data submission from all UK transplant centres for every solid organ transplant performed.
2. *National Cancer Registration and Analysis Service (NCRAS)*. Previously managed by Public Health England (now NHS England), this registry collects data on all individuals diagnosed with cancer in England. It provides comprehensive details on cancer diagnoses, including date of diagnosis, tumour site, histology, and treatment. NCRAS is estimated to capture 98–99% of all cancer cases in England, with the remaining 1–2% comprising patients receiving private treatment or those who have opted out of cancer registration [9].
3. *Civil registration of deaths*. Previously managed by NHS Digital (now NHS England), this dataset contains official records of all deaths in England including date and cause of death as coded using ICD-9 or ICD-10 classifications.
4. *Hospital episode statistics (HES)*. Previously managed by NHS Digital (now NHS England), this administrative database includes records of all secondary care episodes in England, encompassing both inpatient admissions and outpatient appointments (using ICD-9 or ICD-10 classifications) and all surgical operations, procedures and interventions (using Office of Population Censuses and Surveys Classification of Interventions and Procedures [OPCS-4]).

### Linkage methodology

UKTR and NCRAS transferred data to NHS Digital, who identified common patient identifiers within both datasets. NHS Digital extracted the corresponding patients from the Civil Registration of Deaths and HES datasets. A unique pseudonymous ID number was generated for each patient, with the removal of patient identifiable data, and returned to each organisation. Therefore, the EpCOT dataset received at the University of Birmingham was anonymised, linked via the pseudonymous IDs with no patient identifiable information.

### Patient population

This study focussed on de novo cancer only. Recipients were excluded if they ever had a registered cancer prior to transplantation, if cancer was the indication for transplantation (e.g. hepatocellular carcinoma for liver transplantation), or if they were diagnosed with cancer within 30 days post-transplantation, suggesting pre-existing malignancy. Only first transplants were included and follow-up started 30 days post-transplant and continued until cancer-related death, death from another cause, or censoring on 31^st^ December 2022. Non-melanoma skin cancers (NMSCs) were excluded from this analysis. While NMSCs are common among transplant recipients, they are routinely noted to be underreported with significant variability in documentation practices across England [10]. Only malignant tumours were included (ICD-10: C00-C96—excluding NMSC [C44], ICD-9: 140-209—-excluding NMSC [173]), while in situ or benign tumours (ICD-10: D00-D48, ICD-9: 210-234) were excluded.

HES data were used to identify patient demographic details such as deprivation status.

### Statistical Analysis

Standardised mortality ratios (SMRs) for cancer-related mortality were calculated overall and stratified by sex, transplanted organ, and cancer type. SMRs were derived by dividing the observed number of cancer deaths by the expected number, which was estimated by calculating person-years at risk within the cohort. These were stratified by calendar year, sex and 5-year attained age groups, then multiplied by the corresponding cancer mortality rates in the general population. Ninety-five percent confidence intervals (CIs) for SMRs were calculated under the assumption that cancer mortality followed a Poisson distribution. Absolute excess risks (AERs) per 10,000 person-years (PY) were calculated as observed deaths minus the expected deaths, divided by the total person years at risk multiplied by 10,000. AER represents the average excess number of cancer deaths per 10,000 person-years in the study cohort versus the general population.

A multivariable Poisson regression model, offset by the expected number of deaths, was fitted to assess factors associated with cancer mortality, adjusting for sex, age at transplantation, transplanted organ, ethnicity, deprivation status, attained age and year of transplant. Adjusted incidence rate ratios (IRRs) with 95% CI were reported. Likelihood ratio tests were used to compare nested Poisson regression models and evaluate evidence of heterogeneity or linear trend across categories.

A time-to-event analysis was conducted using competing risks regression based on the Fine and Grey sub-distribution hazard model. This allowed estimation of the cumulative incidence of cancer-related death while accounting for death from other causes as a competing event. Cumulative incidence function (CIF) curves illustrate the probability of cancer death overtime in the presence of the competing risk of non-cancer death.

Statistical significance was defined as a two-sided *p*-value < 0.05. Analyses were performed using Stata SE, version 19.5 (StataCorp, College Station, TX) and R, version 4.4.2. The statistical code may be provided the corresponding author upon reasonable request.

### Approvals for record linkage

This study has institutional (*RRK5471*) and ethical (*15/YH/0320*) approval. It also has section 251 approval from the Confidentiality Advisory Group (*16/CAG/0121*), under Regulation 5 of the Health Service (Control of Patient Information) Regulations 2002, to facilitate the record linkage. The legal basis for data processing is under the National Health Service Act 2006 – section 251 ‘Control of patient information’, General Data Protection Regulation Article 6 (1)(e), and General Data Protection Regulation Article 9 (2) (j). The legal basis for dissemination is under the Health and Social Care Act 2012 section 261(1) and section 261(2)(b)(ii).

## Results

### Study cohort

The cohort included 50,762 SOT recipients. Most recipients were aged ≥18 years (83.6%) at the time of transplantation, male sex (60.8%) and of White ethnicity (76.1%) as shown in Table 1.

**Table 1:** Characteristics of all first solid organ transplants in England from 1992 to 2015.

	Heart	Heart and lung	Kidney	Liver	Lung	Other/Multivisceral	Pancreas	SPK	Total
Total	3534 (7.0%)	408 (0.8%)	33,362 (65.7%)	8783 (17.3%)	2381 (4.7%)	388 (0.8%)	209 (0.4%)	1697 (3.3%)	50,762 (100.0%)
Recipient sex									
Female	895 (25.3%)	217 (53.2%)	12,731 (38.2%)	3999 (45.5%)	1031 (43.3%)	184 (47.4%)	139 (66.5%)	698 (41.1%)	19,894 (39.2%)
Male	2639 (74.7%)	191 (46.8%)	20,631 (61.8%)	4784 (54.5%)	1350 (56.7%)	204 (52.6%)	70 (33.5%)	999 (58.9%)	30,868 (60.8%)
Recipient age									
0–17	580 (16.4%)	60 (14.7%)	2,137 (6.4%)	1242 (14.1%)	103 (4.3%)	136 (35.1%)	0 (0.0%)	4 (0.2%)	4262 (8.4%)
18–35	484 (13.7%)	175 (42.9%)	6961 (20.9%)	1223 (13.9%)	443 (18.6%)	64 (16.5%)	40 (19.1%)	436 (25.7%)	9826 (19.4%)
36–50	964 (27.3%)	133 (32.6%)	10,614 (31.8%)	2553 (29.1%)	596 (25.0%)	80 (20.6%)	108 (51.7%)	996 (58.7%)	16,044 (31.6%)
51–65	1477 (41.8%)	40 (9.8%)	10,585 (31.7%)	3344 (38.1%)	1207 (50.7%)	102 (26.3%)	57 (27.3%)	258 (15.2%)	17,070 (33.6%)
>65	29 (0.8%)	0 (0.0%)	3,065 (9.2%)	421 (4.8%)	32 (1.3%)	6 (1.5%)	4 (1.9%)	3 (0.2%)	3560 (7.0%)
Recipient ethnicity									
South Asian	223 (6.3%)	17 (4.2%)	3858 (11.6%)	823 (9.4%)	50 (2.1%)	37 (9.5%)	3 (1.4%)	98 (5.8%)	5109 (10.1%)
Black	68 (1.9%)	1 (0.2%)	1936 (5.8%)	281 (3.2%)	23 (1.0%)	10 (2.6%)	3 (1.4%)	60 (3.5%)	2382 (4.7%)
Chinese/East Asian	10 (0.3%)	0 (0.0%)	269 (0.8%)	33 (0.4%)	2 (0.1%)	1 (0.3%)	0 (0.0%)	5 (0.3%)	320 (0.6%)
Mixed	14 (0.4%)	0 (0.0%)	104 (0.3%)	16 (0.2%)	1 (0.0%)	1 (0.3%)	0 (0.0%)	3 (0.2%)	139 (0.3%)
Other	26 (0.7%)	3 (0.7%)	367 (1.1%)	104 (1.2%)	5 (0.2%)	4 (1.0%)	2 (1.0%)	13 (0.8%)	524 (1.0%)
Unknown	468 (13.2%)	77 (18.9%)	2,604 (7.8%)	316 (3.6%)	171 (7.2%)	7 (1.8%)	2 (1.0%)	36 (2.1%)	3681 (7.3%)
White	2725 (77.1%)	310 (76.0%)	24,224 (72.6%)	7210 (82.1%)	2129 (89.4%)	328 (84.5%)	199 (95.2%)	1482 (87.3%)	38,607 (76.1%)
Index of multiple deprivation									
1 - Most deprived	623 (17.6%)	50 (12.3%)	6761 (20.3%)	1793 (20.4%)	370 (15.5%)	64 (16.5%)	29 (13.9%)	287 (16.9%)	9977 (19.7%)
2	723 (20.5%)	61 (15.0%)	7078 (21.2%)	1806 (20.6%)	472 (19.8%)	90 (23.2%)	44 (21.1%)	410 (24.2%)	10,684 (21.0%)
3	664 (18.8%)	82 (20.1%)	6897 (20.7%)	1689 (19.2%)	516 (21.7%)	75 (19.3%)	51 (24.4%)	398 (23.5%)	10,372 (20.4%)
4	653 (18.5%)	74 (18.1%)	6177 (18.5%)	1521 (17.3%)	481 (20.2%)	80 (20.6%)	47 (22.5%)	288 (17.0%)	9321 (18.4%)
5 - Least deprived	517 (14.6%)	69 (16.9%)	4930 (14.8%)	1141 (13.0%)	338 (14.2%)	56 (14.4%)	30 (14.4%)	258 (15.2%)	7339 (14.5%)
Unknown	354 (10.0%)	72 (17.6%)	1,519 (4.6%)	833 (9.5%)	204 (8.6%)	23 (5.9%)	8 (3.8%)	56 (3.3%)	3,069 (6.0%)
Transplant year									
1992–1997	1149 (32.5%)	181 (44.4%)	5717 (17.1%)	1736 (19.8%)	429 (18.0%)	47 (12.1%)	5 (2.4%)	64 (3.8%)	9328 (18.4%)
1998–2003	926 (26.2%)	171 (41.9%)	6821 (20.4%)	2229 (25.4%)	472 (19.8%)	80 (20.6%)	9 (4.3%)	167 (9.8%)	10,875 (21.4%)
2004–2009	691 (19.6%)	32 (7.8%)	8884 (26.6%)	2175 (24.8%)	598 (25.1%)	108 (27.8%)	62 (29.7%)	647 (38.1%)	13,197 (26.0%)
2010–2015	768 (21.7%)	24 (5.9%)	11,940 (35.8%)	2643 (30.1%)	882 (37.0%)	153 (39.4%)	133 (63.6%)	819 (48.3%)	17,362 (34.2%)

Characteristics of 50, 762 solid organ transplant recipients in England, between 1st June 1992 and 31st December 2015.

*SPK* simultaneous pancreas and kidney transplant.

### Cancer-related mortality

**Fig. 1:**
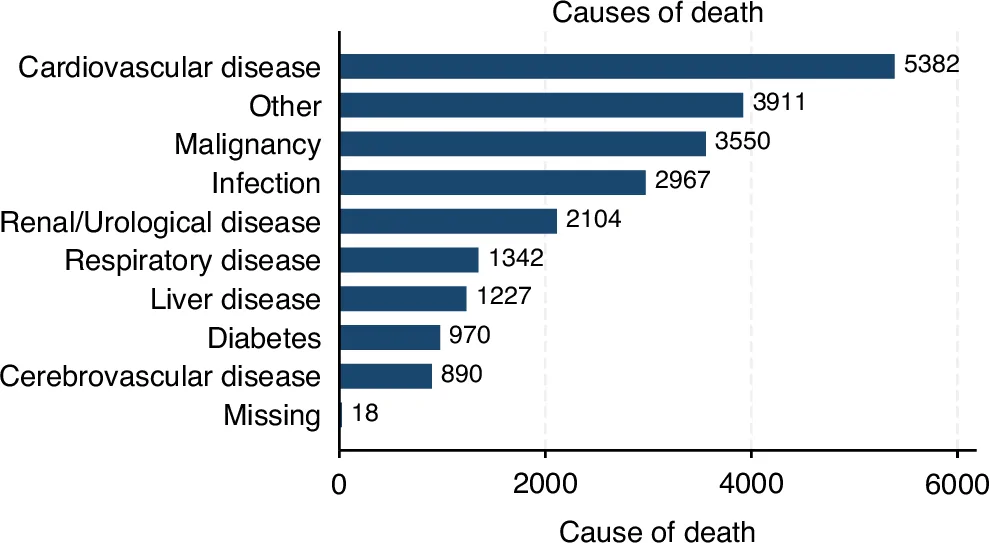
Distribution of underlying causes of death among solid organ transplant (SOT) recipients in England between 1st June 1992 and 31st December 2022 (*N* = 22, 361 deaths). 18 patients had missing ICD-9/ICD-10 codes for cause of death. See supplementary Table 1 for corresponding ICD9/10 codes for cause of death and Supplementary Table 2 for a breakdown of “Other” causes of death.

Across the study period, the cohort contributed 645,649 person-years at risk with a median follow-up of 11.8 years (interquartile range: 7.9–17.5). A total of 22,361 deaths occurred, with 3550 (15.9%) due to cancer, which made cancer the second commonest cause of death after cardiovascular disease (Fig. 1). The leading cause of cancer-related death was lung cancer (676 deaths, 19.0%), followed by non-Hodgkin’s lymphoma (NHL, 490 deaths, 13.8%).

**Fig. 2:**
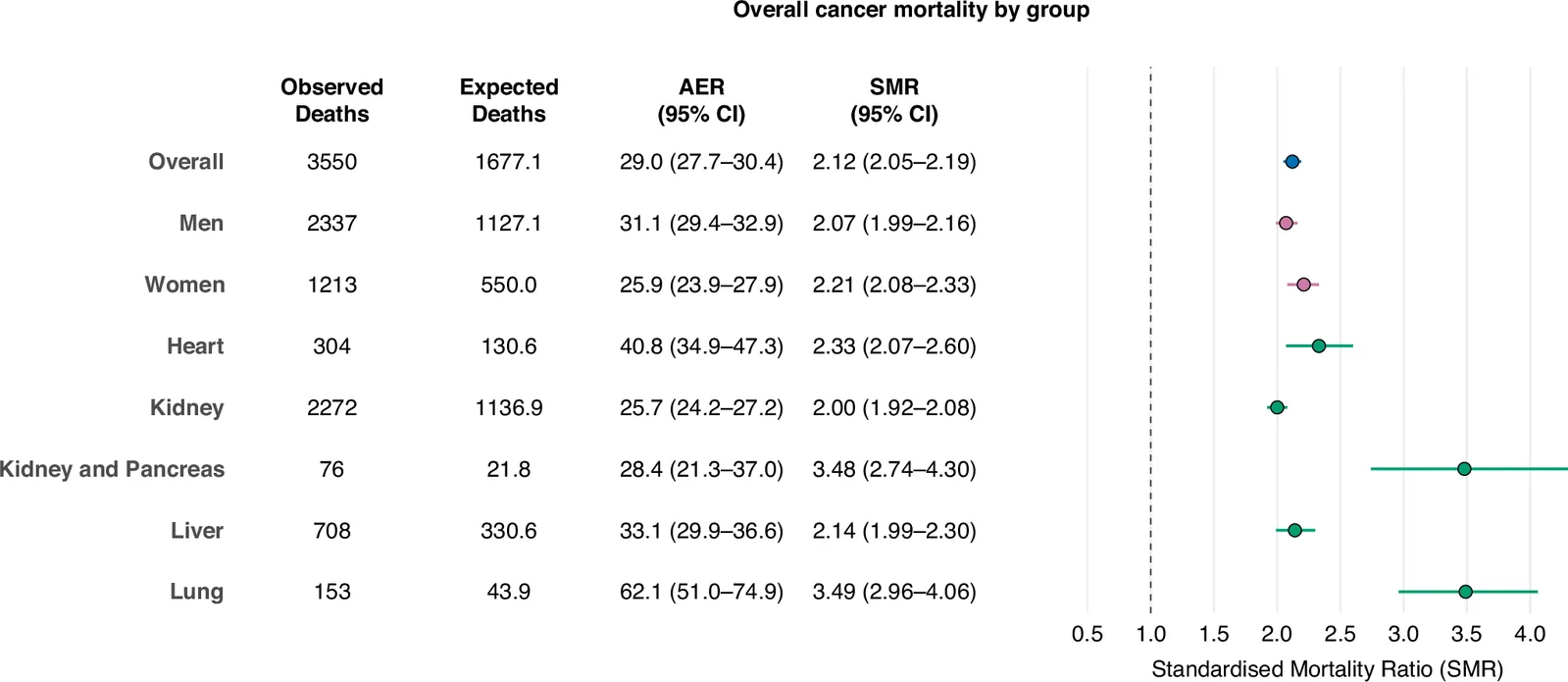
Absolute excess risk (AER) and standardised mortality ratios (SMR) for cancer mortality, stratified by sex and transplant type, with 95% confidence intervals. The dashed line indicates SMR = 1 (no increased risk). AER per 10,000 person years. Abbreviations: CI confidence interval

Overall, SOT recipients had approximately twofold higher cancer mortality than the general population (SMR 2.12; 95% CI, 2.05–2.19), corresponding to 29 additional deaths per 10,000PY of follow-up (95% CI, 27.7–30.4). The SMR was slightly higher in women (2.21; 95% CI, 2.08–2.33) than in men (2.07; 95% CI, 1.99–2.16). Among transplant types with at least 50 cancer-related deaths, the highest mortality was observed in lung transplant recipients (SMR 3.49; 95% CI, 2.96–4.06; AER 62.1 per 10,000 PY; 95% CI, 51.0–74.9), while kidney transplant recipients had the lowest mortality risk (SMR 2.00; 95% CI, 1.92–2.08; AER 25.7 per 10,000 PY; 95% CI, 21.3–37.0) (Fig. 2).

**Fig. 3:**
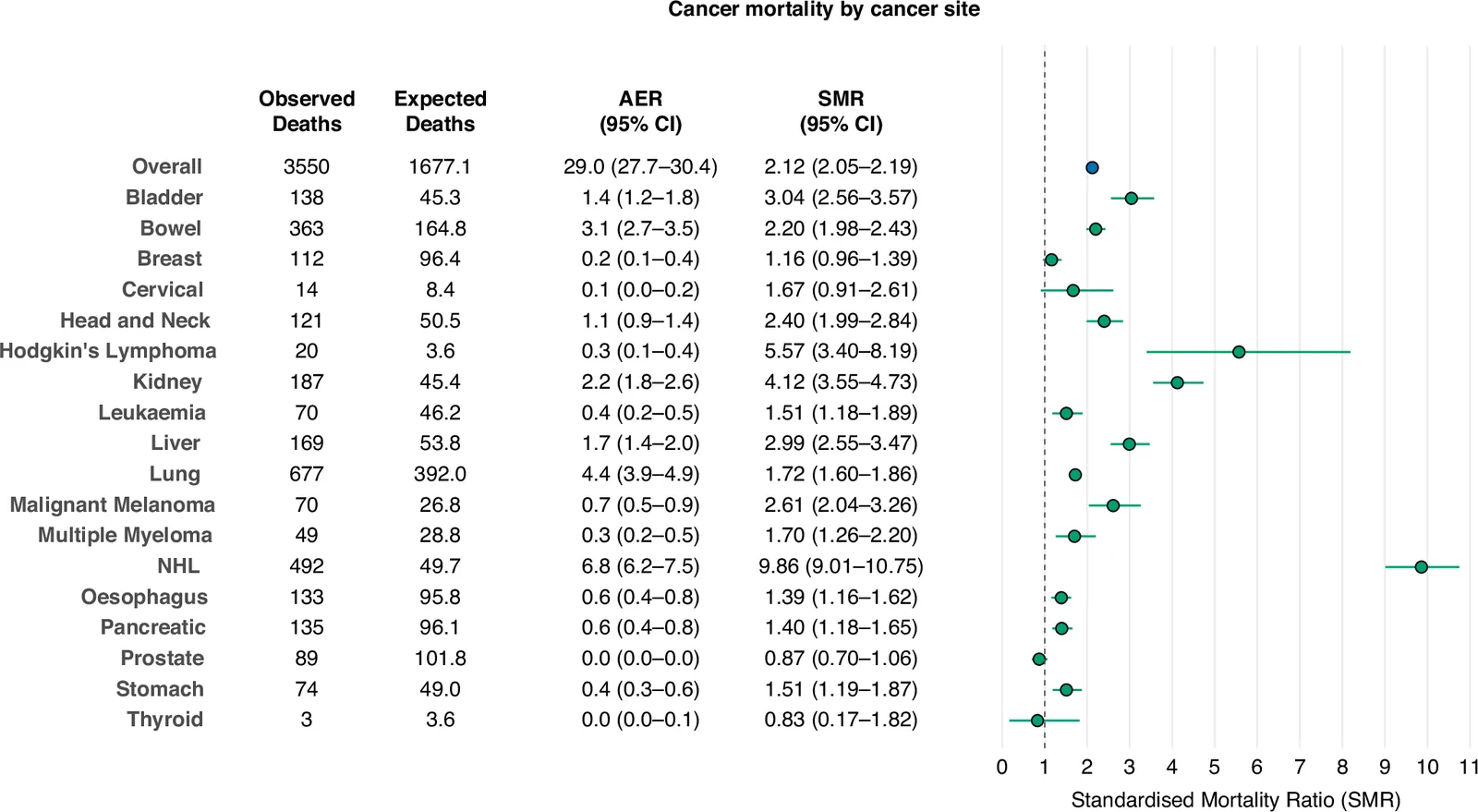
Absolute excess risk (AER) of cancer mortality and standardised mortality ratios (SMR) with 95% confidence intervals for site-specific cancers in the cohort. The vertical dashed line indicates SMR = 1 (no excess risk). AER per 10,000 person years. Abbreviations: CI confidence interval, NHL non-Hodgkin’s lymphoma, Other deaths (*N* = 634): Bone and soft tissue cancer (*n* = 47), central nervous system cancer (*n* = 52), Cancer of unknown primary (*n* = 443), Kaposi’s Sarcoma (*n* = 5), male genital cancer (*n* = 3), gynaecological cancer (*n* = 76), heart, mediastinum or pleural cancer (*n* = 2), parathyroid cancer (*n* = 1), adrenal cancer (*n* = 2), other lymphoid or haematopoietic cancer (*n* = 3).

Cancer-related mortality varied substantially by cancer site. NHL showed the highest mortality with an SMR 9.86 (95% CI: 9.01–10.75) and an AER of 6.8 per 10,000 PY (95% CI 6.2–7.5). In contrast, thyroid cancer (SMR 0.83; 95% CI: 0.17–1.82), prostate cancer (SMR 0.87; 95% CI: 0.70–1.06), cervical cancer (SMR 1.67; 95% 0.91–2.61) and breast cancer (SMR 1.16; 95% CI: 0.91–1.39) did not show increased mortality risk versus the general population (Fig. 3).

### Allograft sub-group analyses

Stratification by both transplant and cancer type revealed further heterogeneity (Supplementary Figs. 1–4). Some cancer-related mortality was elevated across all transplant groups, such as NHL, bowel cancer, and lung cancer. However, organ-specific patterns were also observed. Kidney cancer mortality was markedly elevated among kidney transplant recipients (SMR: 5.11; 95% CI: 4.34–5.93, AER 2.9 per 10,000 PY; 95% CI 2.4–3.4), while liver cancer mortality was only significantly elevated among liver transplant recipients (SMR: 9.30; 95% CI: 7.53-11.24, AER 9.3 per 10,000 PY; 95% CI 6.0-9.3). Importantly, liver cancers were the primary driver of the overall elevated cancer mortality after liver transplantation. When liver cancer deaths were excluded, the overall cancer mortality in this group dropped substantially to an SMR of 1.84 (95% CI: 1.71–2.00), and AER of 24. per 10,000 PY (95% CI 21.9–27.7) placing it below kidney transplant recipients.

### Adjusted analyses

Table 2 presents the adjusted incidence risk ratios (IRRs) for cancer-related death from the multivariable Poisson regression model. There was no significant difference in cancer mortality risk between men and women. However, ethnicity was significantly associated with cancer mortality with South Asian (IRR: 0.59, 95% CI 0.51–0.69), Black (IRR: 0.56, 95% CI 0.45–0.70), and Chinese/East Asian (IRR: 0.55, 95% CI 0.30–0.99) SOT recipients having a lower cancer-related mortality compared to White recipients. Relative to kidney transplant recipients, heart (IRR: 1.22, 95% CI 1.08–1.38), lung (IRR: 1.68, 95% CI 1.42–1.98), and liver (IRR: 1.16; 95% CI 1.06–1.26) transplant recipients had significantly higher risks of cancer-related death, whilst simultaneous kidney-pancreas recipients showed no difference. Patients in the most deprived socioeconomic group experienced a significantly higher risk of cancer-related death compared to those in less deprived groups. Finally, more contemporary SOT recipients demonstrated an increased risk of cancer-related death compared to those transplanted between 1992 and 1997.

**Table 2:** Incidence risk ratio for cancer mortality.

	Incidence risk ratio (95% Confidence Intervals)
Sex	
Female	0.98 (0.92–1.05)
Male	1^a^
P_heterogeneity_	0.582
Recipient ethnicity	
Asian	0.59 (0.51–0.69)
Black	0.56 (0.45–0.70)
Chinese/Oriental	0.55 (0.30–0.99)
Mixed	1.21 (0.54–2.70)
Other	0.53 (0.33–0.84)
White	1^a^
* P*_heterogeneity_	<0.001
Transplanted organ	
Heart	1.22 (1.08–1.38)
Heart and lung	0.71 (0.35–1.41)
Kidney	1^a^
Kidney and Pancreas	1.16 (0.92–1.42)
Liver	1.16 (1.06–1.26)
Lung	1.68 (1.42–1.98)
Other or Multivisceral	1.57 (1.01–2.44)
Pancreas	1.07 (0.55–2.06)
*P*_heterogeneity_	<0.001
Index of multiple deprivation	
1 - Most deprived	1^a^
2	0.85 (0.77–0.95)
3	0.81 (0.72–0.89)
4	0.75 (0.68–0.85)
5 - Least deprived	0.76 (0.68–0.85)
*P*_trend_	<0.001
Year of transplant	
1992–1997	1^a^
1998–2003	1.24 (1.12–1.37)
2004–2009	1.32 (1.18–1.48)
2010–2015	1.32 (1.18–1.49)
*P*_trend_	<0.001

Incidence risk ratios (IRRs) for cancer mortality estimated using a Poisson regression model, adjusted for age at transplantation, sex, ethnicity, deprivation status, transplanted organ and year of transplant.

^a^Reference category.

### Competing risk analyses

In a competing risks regression model, liver transplant recipients had a higher sub-distribution hazard of cancer-related death compared with kidney-only recipients (sub-hazard ratio [SHR] 1.17, 95% CI: 1.07–1.27, *p *= 0.001), whereas simultaneous kidney-pancreas (SHR = 0.79, 95% CI: 0.63–0.99, *p* = 0.048) and lung transplant recipients (SHR = 0.75, 95% CI: 0.63–0.90, *p* = 0.002) had lower risk. No difference was observed for heart transplant recipients (SHR = 1.01, 95% CI: 0.89–1.16, *p* = 0.830). South Asian (SHR = 0.59, *p* < 0.001), Black (SHR = 0.57, *p* < 0.001), and Chinese/East Asian (SHR = 0.54, *p* = 0.046) recipients had significantly lower risk versus White recipients. Socioeconomic deprivation was not associated with cancer mortality (all *p* > 0.15).

**Fig. 4:**
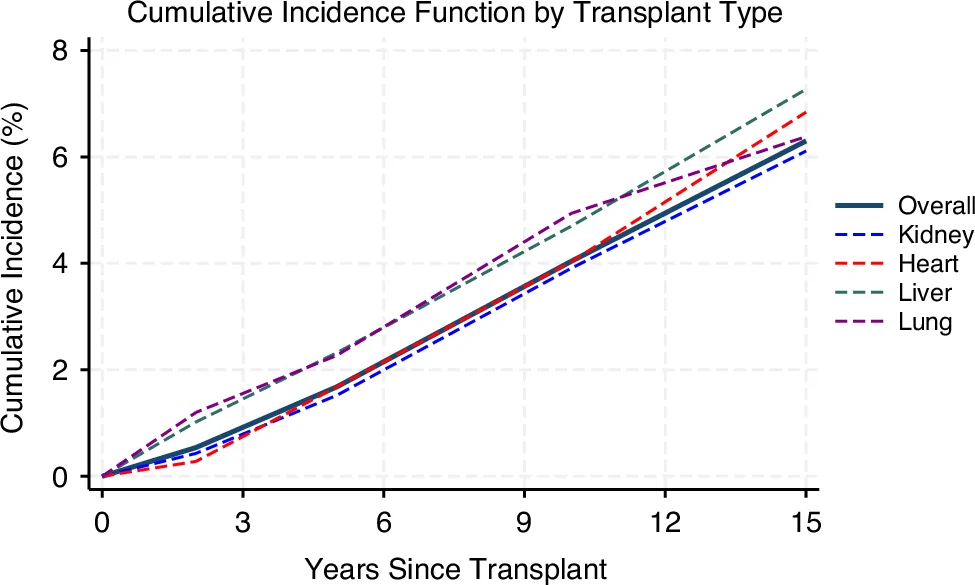
Cumulative incidence of cancer death by transplant type. Cumulative incidence function (CIF) curves showing the probability of cancer death over time, accounting for the competing risk of non-cancer death, stratified by transplant type.

The CIF (Fig. 4) illustrates the probability of cancer-related death over time, considering death from other causes.

## Discussion

This study demonstrated a greater than twofold increase in de novo cancer-related mortality in England versus the general population. Cancer was the second leading cause of death after cardiovascular disease and accounted for 15.9% of all deaths. However, we observed significant variation by cancer site, organ allograft type and recipient characteristics. Our results provide a timely update to current understanding of cancer epidemiology after SOT, with some novel observations that warrant further discussion and targeted investigation.

The primary findings of this study are consistent with previous international studies reporting elevated post-transplantation cancer mortality, with SMRs ranging from 1.4 to 2.9. Our SMR was mid-range of this spectrum but notably higher than the SMR of 1.4 reported by Jeong and colleagues from South Korea [7]. Reasons for this difference are likely multi-factorial. Jeong and colleagues only included kidney transplant recipients, who demonstrated lower cancer-specific mortality in our cohort. Although a similar proportion of patients died from cancer (14.9% in South Korea versus 15.9% in our cohort), the distribution of cancer deaths differed. Liver cancer was the leading cause of cancer death in South Korea (14.6% of cancer deaths), but accounted for only 4.5% in our SOT cohort and 2.2% among kidney recipients. The higher liver cancer burden likely reflects substantially greater prevalence of viral hepatitis in South Korea versus England [11, 12]. South Korea also has lower rates of obesity [13], smoking [14] and spends more per capita on cancer care in comparison to the UK [15], which will influence lower population-level (and SOT) cancer mortality [15]. Country-level differences in demographics, access to healthcare, burden of comorbidities and transplant management underscore the importance of country-specific analyses of cancer mortality after transplantation. Our study therefore, adds importance England-specific population-based evidence that complements studies from other countries.

NHL demonstrated the highest excess cancer-mortality after transplantation, aligning with existing literature [16–18]. NHL after SOT is defined interchangeably with post-transplantation lymphoproliferative disorder (PTLD), which encompasses a spectrum of conditions that manifest from an abnormal proliferation of lymphoid cells (usually B cells) post-transplantation that includes but is not exclusive to NHL [19]. Despite this, screening and/or management recommendations globally are heterogeneous. For example, Epstein–Barr virus (EBV) surveillance is recommended in guidelines from the American Society of Transplantation for adult SOT recipients receiving organs for EBV-seropositive donors [20], but not in British Transplantation Society guidelines [21]. Applying the observed AER of 6.8 per 10,000 PY to accrued person-time indicates approximately 19 excess deaths due to NHL per year in England. This reinforces the need to determine optimal strategies for PTLD with minimised variation in care.

Some cancers had similar mortality risk versus the general population. For example, we did not observe an increased mortality for prostate cancer, which aligns with published literature [8, 18, 22–24]. Prostate carcinogenesis is driven by androgens and growth factors [25–27], with no established association with oncogenic viruses, and is considered an immunologically ‘cold’ tumour with limited immune-cell infiltration[28]. This contributes to its poor responsiveness to immune checkpoint inhibitors and likely lack of influence from immunosuppression. Additionally, transplant recipients are subject to regular and lifelong specialist follow-up and monitoring which, combined with the ease of prostate-specific antigen (PSA) testing, may facilitate earlier detection and treatment. Likewise, for breast and cervical cancer, both part of national screening strategies, we did not observe any increased risk for cancer-related mortality. This would suggest that SOT recipients do not require any enhanced screening due to their transplant status, which aligns with cancer screening recommendations after transplantation [29]. The exception was bowel cancer, which had higher cancer-related mortality. Several factors may account for this observation. Primary sclerosing cholangitis accounts for 13.8% of liver transplants performed in the United Kingdom [30] and carries a 7–10 fold increased risk of bowel cancer owing to its strong association with inflammatory bowel disease [31, 32]. Obesity, a leading factor in end-organ failure [33, 34], is associated with colorectal cancer [35] and diabetes represents an additional risk factor [36]. There is some evidence that immunosuppression influences bowel carcinogenesis unlike prostate or breast carcinogenesis [37]. Better screening adherence could be hypothesised, which may also explain why cervical cancer-mortality rates (despite having an oncogenic pathophysiology) are no different between transplant recipients and the general population. However, a study from Ontario [38] demonstrated 77.5%, 69.8% and 91.4% of SOT recipients were not up-to-date with their recommended bowel, cervical and breast cancer screening, respectively, during the observation period. These rates are likely an overestimate due to the inability to differentiate between screening versus diagnostic tests. Therefore, rather than more frequent cancer screening, SOT recipients simply require encouragement to attend their routine invitations.

By contrast, we noted increased organ-specific risks for certain cancers. For example, liver cancer mortality was significantly elevated among liver transplant recipients, while kidney cancer mortality was significantly elevated among kidney transplant recipients. The mechanisms for this are likely to be very different. In liver transplant recipients, the pro-inflammatory role of underlying cirrhosis and its associated risk factors, such as metabolic dysfunction-associated steatotic liver disease, hepatitis C virus infection, and/or obesity, is associated with the pathogenesis of liver cancer [39]. For kidney transplant recipients, duration of pre-transplant dialysis has one of the strongest associations with post-transplant risk for renal cell carcinomas [40] that primarily arise in native kidneys. These organ allograft-specific cancer mortality risks suggest that some routine cancer surveillance in these bespoke settings may be clinically justified but requires evaluation for cost-effectiveness.

Lung transplant recipients demonstrated consistently elevated cancer mortality when assessed using SMRs, AERs and multivariable Poisson regression, compared with kidney transplant recipients, which may reflect a higher burden of immunosuppression given after lung versus kidney transplantation. However, the competing risks model showed a significantly lower sub-distribution hazard for cancer death. This discrepancy likely reflects the high burden of competing mortality in lung recipients: although their cancer-specific mortality rate is elevated, their cumulative probability of dying from cancer is reduced because they are more likely to die from other causes. Infection is a leading early cause of death following lung transplantation, but this group also has the poorest 5-year survival of all graft types; 54.8% in the UK [41] and 59.7% in the United States [42]. Much of this excess mortality is attributable to chronic lung allograft dysfunction, which affects up to 50% of lung transplant recipients within 5 years after transplantation [43]. and is the leading cause of death beyond the first post-operative year [44, 45]. Thus, while lung transplant recipients have higher cancer mortality rates, their cumulative incidence of cancer death is attenuated by competing risks.

SOT recipients in the most deprived socioeconomic group experienced higher rates of cancer mortality, consistent with previous findings [46, 47]. Despite universal health coverage in England, social determinants of health are well acknowledged. This observation may also reflect differences in patient comorbidities, health literacy and/or lifestyle factors (e.g. smoking, poor diet, physical inactivity), which are more prevalent with greater socioeconomic deprivation [48–51]. and associated with cancer. However, competing risk analyses demonstrated no increased probability of cancer death across deprivation categories. Despite higher cancer-specific mortality rates, no higher overall probability of cancer death exists due to a high burden of competing risks for death. Cardiovascular mortality, highest in the most deprived socioeconomic groups in England [52] shares similar lifestyle risk factors with cancer, likely accounts for a substantial proportion of these competing deaths.

We observed lower cancer-related mortality among Black, South Asian, and Chinese recipients compared with White recipients, likely reflecting the lower cancer incidence reported among non-White populations at a UK population level [53]. The underlying mechanism remains unclear but are likely multifactorial, including healthcare access, socioeconomic factors, environmental exposures, and modifiable behavioural risk factors rather than inherent genetic differences.

We observed an increased risk for cancer mortality in recent years, a trend similarly reported in Italy [16]. Improved mortality and graft survival mean recipients are living longer, with increasing cumulative exposure to immunosuppression throughout their life. Secondly, changes in immunosuppression regimens (e.g. transition in the mid-2000s from largely ciclosporin to tacrolimus-based regimens) has been linked to increased post-transplant cancer risk as tacrolimus is more carcinogenic than ciclosporin [54]. Thirdly, with growing physician awareness, there may be more rigorous surveillance and/or improved detection of cancer potentially increasing the reporting of cancer-related deaths. Nonetheless, the rising trend is a concern. Blosser et al. demonstrated survival outcomes among kidney transplant recipients who develop cancer in the United States over the last three decades have not improved apart from PTLD [55]. This could be due to cancer management after SOT complicated by a greater burden of co-morbidities or therapeutic limitations (e.g. rejection risk with concomitant checkpoint inhibitors and immunosuppression) [56].

This study has several strengths. It is the largest UK-based analysis of cancer mortality in SOT recipients, with linked national registry data enabling comprehensive ascertainment and extended follow-up (median 11.8 years), with outcome-specific follow-up determined by registry data availability. Cancer incidence is highest in the early years post-transplant, however extended follow-up captures late malignancies and their contribution to long-term mortality. However, several limitations must be acknowledged. Firstly, we could not account for individual-level factors such as immunosuppression regimens, dialysis vintage, participation in cancer screening programmes, and modifiable behavioural risk factors (e.g. smoking, alcohol, dietary habits). Secondly, NMSC was excluded due to the known underreporting and inconsistent documentation within NCRAS, which may lead to an underestimation of the overall cancer burden in our cohort. While the cancer-specific mortality risk is low with NMSC, data from the general population show an increased risk for all-cause and secondary cancer outcomes for people who develop squamous cell versus basal cell carcinomas [57]. Thirdly, cancer mortality is influenced by cancer incidence and post-diagnosis survival, which should be considered simultaneously when interpreting our findings.

To conclude, cancer remains a major contributor to long-term mortality among SOT recipients in England in the contemporary era of transplantation care. These findings underscore the importance of long-term cancer vigilance and heightened clinical awareness for transplant recipients, particularly for high-risk cancers such as PTLD. While mortality risk for commonly screened cancers is low, and justifies no deviation from standard screening frequency, elevated organ-specific cancer mortality risk (liver or kidney cancer mortality risk for liver or kidney transplant recipients, respectively) warrants further evaluation for efficacy and cost-effectiveness. With improved survival outcomes associated with SOT and mitigation of allograft rejection risk, enhanced focus is required on immunosuppression-related complications, such as cancer to support efforts to prolong longevity and quality of life for SOT recipients and bridge the survival gap with the general population.

## Supplementary information

Supplemental Material
