# Supplementary material for: De novo cancer-related mortality after solid organ transplantation in England: the EpCOT study
Source: Br J Cancer. 2026 Jul 1;135(7):1086–93. doi: 10.1038/s41416-026-03529-4 (PMC13578314; doi:10.1038/s41416-026-03529-4)
Supplement: Supplementary file 1 [file 41416_2026_3529_MOESM1_ESM.docm]

**De Novo Cancer-Related Mortality after Solid Organ transplantation in England: the EpCOT study – Supplement**

Supplement with a breakdown of causes of death for the cohort and forest plots demonstrating the absolute excess risk and standardised mortality ratios of site-specific cancers, broken down by transplant type.

Table S1 – Causes of death for all solid organ transplant recipients in England between 1^st^ June 1992-31^st^ December 1995, with corresponding ICD 9 and ICD 10 codes

| **Cause of Death** | **Number of Deaths** | **ICD 9 codes** | **ICD 10 codes** |
| --- | --- | --- | --- |
| Cardiovascular disease | 5,382 | 393-429  440-459  745-747  785 | I00-I52  I70-I99 |
| Malignancy | 3,550 | 140-172  174-209 | C00-C43  C45-C97 |
| Infection | 2,967 | 000-139  320-326  614-616  380-383  390-392  460-466  480-488  590  566-567  680-686  595  601  730 | A00-B99  G00-G08  H60  H66-H71  J00-J22  J85- J86  K61  K63  K65  K81  L00-L08  M00-M01  M86  N30-N34  N70-N73  U07.1-U10 |
| Renal/Urological disease |  | 580-589  591-594  596-599 | N00-N29  N35-N39  Q61 |
| Respiratory disease | 1,342 | 470-478  490-519 | J30-J84  J90-J99 |
| Liver disease | 1,227 | 570-573 | K70-K77 |
| Diabetes | 970 | 250 | E10-E14 |
| Cerebrovascular disease | 890 | 430-438 | I60-I69 |
| Other | 3,911 | N/A | N/A |
| Missing ICD9/10 code | 18 | N/A | N/A |

Table S2 – Breakdown of the 3,911 “Other” causes of death

| **Cause of Death** | **Frequency** |
| --- | --- |
| Acute appendicitis | 6 |
| Amyloidosis | 51 |
| Benign neoplasm | 12 |
| Biliary tract disease | 208 |
| Chromosomal disorder | 10 |
| Complications of medical or surgical care | 332 |
| Congenital malformation/deformation | 175 |
| Connective tissue disease | 1 |
| Cystic Fibrosis | 329 |
| Dementia | 203 |
| Diverticular disease | 90 |
| ENT disease | 28 |
| Endocrine disease | 13 |
| Epilepsy | 26 |
| Fall | 124 |
| Hernia | 33 |
| Haematological disease | 107 |
| Hypothermia | 7 |
| Inflammatory arthritis | 17 |
| Inflammatory Bowel Disease | 29 |
| Intestinal malabsorption/ malnutrition | 11 |
| Intracranial injury | 11 |
| Ischemic bowel | 207 |
| Mediastinitis | 7 |
| Metabolic disorder | 277 |
| Motor neuron disease | 26 |
| Neoplasm of uncertain/unknown behavior | 169 |
| Non melanomatous skin cancer | 182 |
| Non-inflammatory gynaecological disease | 2 |
| Obesity | 7 |
| Oesophageal disease | 22 |
| Osteonecrosis | 3 |
| Osteoporosis | 19 |
| Other accident | 101 |
| Other arthritis | 13 |
| Other disease of the stomach/intestine/bowel | 55 |
| Other disorder of the male genital organs | 16 |
| Other disorders of the immune system | 42 |
| Other muscle disorder | 34 |
| Other neurological disease | 70 |
| Other skin disorder | 46 |
| Pancreatitis | 124 |
| Parkinsons disease | 30 |
| Perinatal/Neonatal disease | 3 |
| Poisoning | 99 |
| Pregnancy related | 14 |
| Psychiatric disease | 24 |
| Sarcoidosis | 26 |
| Small bowel obstruction | 60 |
| Spina bifida | 22 |
| Suicide | 61 |
| Systemic lupus erythematosis | 49 |
| Systemic Sclerosis | 14 |
| Transport accident | 35 |
| Ulcer – pressure/vascular | 17 |
| Unspecified cause of death | 38 |
| Upper GI haemorrhage | 38 |
| Upper GI ulcer | 81 |
| Vasculitis | 51 |

Figure S1 - Absolute excess risk (AER) of cancer mortality and standardised mortality ratios (SMR) with 95% confidence intervals for site-specific cancers among kidney transplant recipients in the cohort. The vertical dashed line indicates SMR = 1 (no excess risk).

Notes: AER per 10,000 person years
Abbreviations: CI = Confidence interval, NHL = Non-Hodgkin’s Lymphoma
Other deaths (N=438): Bone and Soft tissue cancer (n=29), Central nervous system cancer (n=37), Cancer of unknown primary (n=305), Kaposi’s Sarcoma (n=5), Male genital cancer (n=2), Gynaecological cancer (n=57), Parathyroid cancer (n=1), Adrenal cancer (n=2).

Figure S2 - Absolute excess risk (AER) of cancer mortality and standardised mortality ratios (SMR) with 95% confidence intervals for site-specific cancers among liver transplant recipients in the cohort. The vertical dashed line indicates SMR = 1 (no excess risk).

Notes: AER per 10,000 person years
Abbreviations: CI = Confidence interval, NHL = Non-Hodgkin’s Lymphoma
Other deaths (N=109): Bone and Soft tissue cancer (n=9), Central nervous system cancer (n=10), Cancer of unknown primary (n=70), Male genital cancer (n=1), Gynaecological cancer (n=16), Heart, mediastinum or pleural cancer (n=2), Other lymphoid or haematopoietic cancer (n=1).

Figure S3 - Absolute excess risk (AER) of cancer mortality and standardised mortality ratios (SMR) with 95% confidence intervals for site-specific cancers among heart transplant recipients in the cohort. The vertical dashed line indicates SMR = 1 (no excess risk).

Notes: AER per 10,000 person years
Abbreviations: CI = Confidence interval, NHL = Non-Hodgkin’s Lymphoma
Other deaths (N=45): Bone and Soft tissue cancer (n=5), Central nervous system cancer (n=4), Cancer of unknown primary (n=35), Gynaecological cancer (n=1)

Figure S4 - Absolute excess risk (AER) of cancer mortality and standardised mortality ratios (SMR) with 95% confidence intervals for site-specific cancers among lung transplant recipients in the cohort. The vertical dashed line indicates SMR = 1 (no excess risk).

Notes: AER per 10,000 person years
Abbreviations: CI = Confidence interval, NHL = Non-Hodgkin’s Lymphoma
Other deaths (N=27): Bone and Soft tissue cancer (n=2), Central nervous system cancer (n=1), Cancer of unknown primary (n=22), Other lymphoid or haematopoietic cancer (n=2).

Figure S5 - Kaplan–Meier overall survival curves stratified by transplant type (kidney, heart, liver, and lung). The x-axis represents years since transplantation, and the y-axis represents survival probability. Shaded areas represent 95% confidence intervals.
